# Altered Expression of Wnt Signaling Pathway Components in Osteogenesis of Mesenchymal Stem Cells in Osteoarthritis Patients

**DOI:** 10.1371/journal.pone.0137170

**Published:** 2015-09-09

**Authors:** Pilar Tornero-Esteban, Ascensión Peralta-Sastre, Eva Herranz, Luis Rodríguez-Rodríguez, Arkaitz Mucientes, Lydia Abásolo, Fernando Marco, Benjamín Fernández-Gutiérrez, José Ramón Lamas

**Affiliations:** 1 Instituto de Investigación Sanitaria del Hospital Clínico San Carlos (IdISSC), UGC Reumatología, Hospital Clínico San Carlos, 4ª Planta, Ala Norte, C/ Profesor Martín Lagos s/n, 28040, Madrid, Spain; 2 Instituto de Investigación Sanitaria del Hospital Clínico San Carlos (IdISSC), UGC Traumatología, Hospital Clínico San Carlos, 6ª Planta, Ala Norte, C/ Profesor Martín Lagos s/n, 28040, Madrid, Spain; Georgia Regents University, UNITED STATES

## Abstract

**Introduction:**

Osteoarthritis (OA) is characterized by altered homeostasis of joint cartilage and bone, whose functional properties rely on chondrocytes and osteoblasts, belonging to mesenchymal stem cells (MSCs). WNT signaling acts as a hub integrating and crosstalking with other signaling pathways leading to the regulation of MSC functions. The aim of this study was to evaluate the existence of a differential signaling between Healthy and OA-MSCs during osteogenesis.

**Methods:**

MSCs of seven OA patients and six healthy controls were isolated, characterised and expanded. During *in vitro* osteogenesis, cells were recovered at days 1, 10 and 21. RNA and protein content was obtained. Expression of WNT pathway genes was evaluated using RT-qPCR. Functional studies were also performed to study the MSC osteogenic commitment and functional and post-traslational status of β-catenin and several receptor tyrosine kinases.

**Results:**

Several genes were downregulated in OA-MSCs during osteogenesis *in vitro*. These included soluble Wnts, inhibitors, receptors, co-receptors, several kinases and transcription factors. Basal levels of β-catenin were higher in OA-MSCs, but calcium deposition and expression of osteogenic genes was similar between Healthy and OA-MSCs. Interestingly an increased phosphorylation of p44/42 MAPK (ERK1/2) signaling node was present in OA-MSCs.

**Conclusion:**

Our results point to the existence in OA-MSCs of alterations in expression of Wnt pathway components during *in vitro* osteogenesis that are partially compensated by post-translational mechanisms modulating the function of other pathways. We also point the relevance of other signaling pathways in OA pathophysiology suggesting their role in the maintenance of joint homeostasis through modulation of MSC osteogenic potential.

## Introduction

Osteoarthritis (OA) is the most prevalent form of arthritis. It is a degenerative disease that involves the whole joint structure. Disease process is characterized by cartilage loss and structural changes in bone, including formation of marginal outgrowths, osteophytes and sclerosis. Moreover, affectation of other soft-tissue structures is also present in OA. These include muscle, tendon and ligament weakness as well as symptomatic synovial inflammation.[1, 2] OA incidence, mainly in aging joints and in those suffering from previous injuries or articular stress has suggested the biomechanically-driven nature of this disease, however biochemical and/or genetic alterations are likely involved.[3, 4] Initiation and/or progression of OA has been associated with failure to repair and/or an anomalous remodeling of joint constituents, in particular cartilage and subchondral bone whose mutual interplay is considered essential in disease process [1, 5]

In advanced stages, OA differs from other joint diseases by hypertrophic changes occurring in bone.[6] Besides, OA is associated to increased bone mass and an imbalance between bone formation and bone resorption. [7]

Bone and cartilage function depends on the synthesis and secretion of extracellular matrix (ECM) by two tissue specific cell types: the osteoblasts, and the chondrocytes belonging to the MSC lineage of progenitors.[8] Osteoblasts in addition are required for osteoclast differentiation, and thereby for bone resorption and proper turnover.[9] However, in OA articular cartilage is progressively replaced by bone, resembling the mechanisms occurring during skeletal development by endochondral ossification.[10] Clinical and *in vitro* evidences point to the importance of the Wnt signaling pathway in regulating osteogenesis, skeletal development and its involvement in the OA pathogenesis, as evidenced the deregulation seen for some members of this pathway in OA.[10–12] However, it remains unclear whether disturbances, leading to skeletal dysplasias and articular diseases such as OA, are due to a malfunction of MSCs, responsible for their remodeling, or if the disease itself has detrimental effects affecting the MSCs regenerative capacity. In any case, proper function of Wnt signaling is essential to maintain the homeostasis of articular structures.[13]

The Wnt family of secreted factors regulates the essential developmental processes of cell fate and polarity, as well as other general cell maintenance processes. At least nineteen Wnts, ten Fzd receptors, two co-receptors (LRP-5, LRP-6) and several inhibitors (Dkks, sFrps and Wif) have been described in humans.[14] Wnt signaling includes several pathways.[15] The best characterized is the canonical, or Wnt/β-catenin dependent, that signals through frizzled receptors and LRP-5 or LRP-6 co-receptors promoting the activation of disheveled (DVL), which in turn blocks the function of GSK-3β (glycogen synthase kinase 3β). Subsequent stabilization and cytoplasmic accumulation of β-catenin promotes its translocation to the nucleus where it binds to and activates the TCF/LEF family of transcription factors leading to transcriptional activation of constitutively bound Wnt target genes. Conversely, in the absence of Wnts, β-catenin is phosphorylated, destabilized and degraded by the proteasome. Altogether, these pathways participate in developmental events occurring, not only during early embryogenesis,[16] but also in adult tissue homeostasis [17, 18] triggering pleiotropic effects including the mitogenic stimulation, tropism, cell fate commitment, cell differentiation and apoptosis.[19, 20] In addition MSCs also contribute to reduce the inflammatory and immune reactions.[21, 22]

Homeostatic maintenance of articular structures results from a fine-tuned crosstalk between pathways regulating essential cell functions, in particular the Wnt and other collateral signaling pathways.[23] Although the complexity of this regulation needs further research to outline the mechanisms occurring during osteogenesis and in dysregulated processes in bone related pathologies is essential to develop new strategies to improve therapeutic applications of MSC based therapies. The aim of this study was to evaluate comparatively the existence and association of differences in signaling networks in OA derived MSCs and healthy MSCs.

## Materials and Methods

### Patients

Main experiments were performed using bone marrow obtained from the femoral head at the time of surgery for total hip replacement of seven patients with osteoarthritis (six males and one female. Median age 77 years, range [59–82]) and six healthy donors suffering traumatic subcapital fracture (one male and five females. Median age 82 years, range[64–92]), OA diagnosis was established according to the American College of Rheumatology criteria. Healthy donors did not show radiographic changes of OA or osteoporosis (densitometric T-score >-2.5SD). Radiographic changes were evaluated by an independent observer to asses a clear distinction between OA and non-OA subjects. For quantitation of β-catenin, additional MSCs samples were obtained from eight healthy and six OA donors respectively. Written informed consent was obtained from all patients before sample collection. The study was approved following the guidelines of the institutional ethics committee (Comité Ético de Investigación Clínica Hospital Clínico San Carlos–Madrid) and the principles expressed in the Declaration of Helsinki.

### Cell Cultures

BM-MSCs (hereinafter referred to as MSCs) were prepared from bone marrow aspirates of patients undergoing surgery for total hip replacement. Briefly, aspirates were diluted with an equal volume of saline and centrifuged over ficoll at 2,000 x g for 20 min. Cellular fraction was recovered and washed twice in Dulbecco’s Modified Eagles Medium (DMEM) (Sigma). Cell pellet was resuspended in 5ml with culture medium (DMEM supplemented with 2mM glutamine, 0.06% penicillin, 0.02% streptomycin and 10% FBS). Cultures were maintained at 37°C in a humidified atmosphere containing 5% CO_2_. A confluent monolayer of adherent cells, in 25 cm^2^ flasks, was obtained after refreshing the culture medium every 2 days. Cells were passaged two more times to obtain sufficient amount of cells and then cryopreserved for later analysis. All cultures from individual donors were maintained separately.

Proliferation of MSCs was calculated analyzing the population doubling (PD) curve of expanded MSCs. The number of MSCs at each passage was manually counted and the PD for each passage was calculated using equation: N = log2 (Nh/Ni) where N = population doublings, Nh = cell harvest number, Ni = plating cell number, and the cumulative PDs were calculated in relation to the number of cells at the first passage.

### Flow Cytometry

Immunophenotyping was performed on cell cultures recovered from cryopreserved stocks at the third passage. Cells were grown to confluence and expression of surface markers were evaluated by flow cytometry, according to the ISCT criteria [24]. Antibodies used were as follows: mouse anti-human IgG1 against CD73, CD90 and CD105, CD14, CD34 and (rat anti-human IgG2b) CD45. All the antibodies and isotype controls were R-phycoerytrin (PE) conjugated (Miltenyi Biotech). Immunostaining was performed incubating for 30 minutes at 4°C. After washing, cells were fixed with 0.1% paraformaldehyde prior to analysis in a “Gallios” flow cytometer (Beckman Coulter).

### Differentiation Experiments and Histochemistry

Cells were grown from cryopreserved stocks to confluence in twelve-well-plates and then subjected to specific differentiating conditions using the osteogenic differentiation medium PT-4120, and chondrogenic medium PT-3003 supplemented with 10 ng/mL TGFβ3, PT-4124 (all from Lonza) under conditions described by the manufacturer.

Multilineage differentiation potential of MSCs was assessed by histochemical staining and phase contrast microscopy (Leica 4000b DMI, Leica Microsystems GmbH. Germany).

The degree of mineralization in osteogenic cultures was assessed staining with 2% Alizarin Red S (Sigma-Aldrich-Aldrich). Briefly, cells were washed three times with phosphate buffered saline (PBS) pH 4.2 and fixed 20 minutes with 4% paraformaldehyde. Fixed cells were washed and stained and washed again to remove excess of stain. Similarly, chondrogenic potential was evaluated measuring production ECM proteoglycan produced by chondrocytes after staining for 20 minutes with 1% Alcian blue (Sigma-Aldrich).

### Induction of Osteogenic Differentiation

MSCs were seeded at 2.5×10^3^ cells/cm^2^ and cultured in PT-4120 osteogenic medium (Lonza) in six well plates changing the osteogenic medium every 3 days. Control cultures were performed with the same medium without osteogenic supplements. RNA and protein content was recovered at days 1, 10 and 21 for further analyses. Quantitative analysis of Alizarin Red Staining was performed after dye extraction with 200 μL 10% acetic acid during 30 minutes under agitation. Recovered supernatant was further centrifuged, heated to 85°C for 10 minutes and cooled at 4°C. After centrifugation at 20,000xg for 15 minutes pH of supernatant was neutralized using 75 μL of 10% Ammonium hydroxide. Concentrations were calculated by determining OD^405^ against known Alizarin Red concentrations.

### Human Wnt Signaling Pathway PCR Array

RNA was isolated from RNAlater conserved samples using the QIAMP RNA miniKit (Qiagen) and retrotranscribed using the RT^2^ amplification Kit (SABiosciences), following the manufacturer´s instructions. The expression of 84 genes related to Wnt-mediated signal transduction was performed using the Human Wnt signaling pathway RT² Profiler PCR Array PAHS-043A, (SABiosciences) according to manufacturer's protocol in a Mastercycler ep Realplex^4^ S (Eppendorf, Germany). Five OA donors and three healthy donors were used in these experiments.

Quantitative RT-PCR was performed and relative gene expression for each gene was calculated using the 2^−ΔΔCt^ method. ΔCt for each sample was normalized using the geometric average of *RPL13A* gene Ct values, proven to be the most stable across samples. Detectable PCR products were obtained and only Ct values > 35 cycles were considered inespecific and discarded for further calculations.

Raw data were then analyzed using the SABioscience web-based PCR Array Data Analysis tool (http://www.sabiosciences.com/pcrarraydataanalysis.php). Fold change values were expressed as 2^−ΔΔCt^ for genes in OA MSCs relative to Healthy-MSCs.

### Quantitative PCR of Osteogenic Lineage Specific Genes

To perform quantitative the RT–PCR analyses, cDNA was first synthesized from mRNA (1 μg) using the Maxima H minus First Strand cDNA Synthesis Kit (Thermo Scientific). PCR amplification reactions were performed in a Mastercycler realplex4 (Eppendorf) using the MAXIMA SYBR Green/ROX Master Mix (Thermo Scientific). After an initial step of 95°C for 10 min, the following cycling conditions were used: 40 cycles of 95°C for 15 s; and 60°C for 1 min. Forward and Reverse primer sequences (5’>3’) were: Alkaline Phosphatase (ALP) Forward: ATT TCT CTT GGG CAG GCA GAG AGT; Reverse: ATC CAG AAT GTT CCA CGG AGG CTT. Osteopontin (OPN); Forward: AGA ATG CTG TGT CCT CTG AAG; Reverse: GTT CGA GTC AAT GGA GTC CTG. Osteocalcin (BGLAP): Forward: CAG CGA GGT AGT GAA GAG AC; Reverse: TGA AAG CCG ATG TGG TCA G; GAPDH: Forward: TTC GAC AGT CAG CCG CAT CTT CTT, Reverse: GCC CAA TAC GAC CAA ATC CGT TGA; RUNX2; Forward: GGA GTG GAC GAG GCA AGA GTT T, Reverse: AGC TTC TGT CTG TGC CTT CTG G. All reactions were done by duplicate and quantitation was calculated using the ΔΔCt method.

### Phospho-Protein Array Analysis

Osteogenic cultures were harvested at days 1, 10 and 21 and cell lysates were obtained using the Cell Lysis Buffer provided in the PathScan RTK Signaling Antibody Array Kit (Cat#: 7982. Cell Signaling. Danvers, MA, USA) (S1 Table) Protein extracts were used to perform a slide-based antibody array according to the manufacturer recommended protocol. Briefly, 250 μg of protein from each cell lysate was applied to a pre-blocked antibody array and incubated overnight at 4°C with constant rocking. The arrays were washed three times for 5 minutes and serially incubated for 1 hour with the biotinylated detection antibody cocktail and for 30 min with HRP-linked Streptavidin. After further washing, the arrays were incubated with LumiGLO/hydrogen peroxide reagent. Slide images were immediately captured using a digital imaging system for chemiluminescent signals (Alliance 4.7 UVItec, UK). Duplicate spot intensities were quantified from each array image using the public software package ImageJ v1.45s.

### Total β-Catenin Enzyme-Linked Immunosorbent Assay (ELISA)

These experiments were performed using different MSC samples, including eight new healthy and six OA donors. Lysates of 1.5 x 10^6^ MSCs were isolated for total β-Catenin analysis. Briefly, cells were treated with cell lysis buffer (Cell signaling, Cat. #9803) supplemented with 1mM PMSF and protease inhibitor cocktail (Sigma Cat.#P8340). Protein extracts were further quantified using the BCA Protein assay Kit (Thermo Scientific, Cat. #23250). 10μg of protein extracts were assayed using the phosphoELISA Kit (Cat. #KHO1211, Invitrogen) for determination of total β-Catenin content of lysates, following manufacturer instructions.

### Statistical Analysis

The p values were calculated based on a Student’s t-test of replicate Ct values. Fold change (2^–ΔΔCt^) represented the normalized gene expression (2^–ΔCt^) in the Test Sample divided the normalized gene expression (2^–ΔCt^) in the Control Sample. The fold-regulation represent the fold-change results in a biologically meaningful way, in our study a significance threshold expressed (>2.5-fold) was considered. Fold-changes greater than 2.5 or less than -2.5 indicate respectively the gene up-regulation or down-regulation in OA patients compared with controls. Unpaired data were analyzed using the nonparametric Mann-Whitney U test. p<0.05 values were considered statistically significant.

## Results

### Phenotypic and Functional Characterization of MSCs from OA and Control Samples

**Fig 1:**
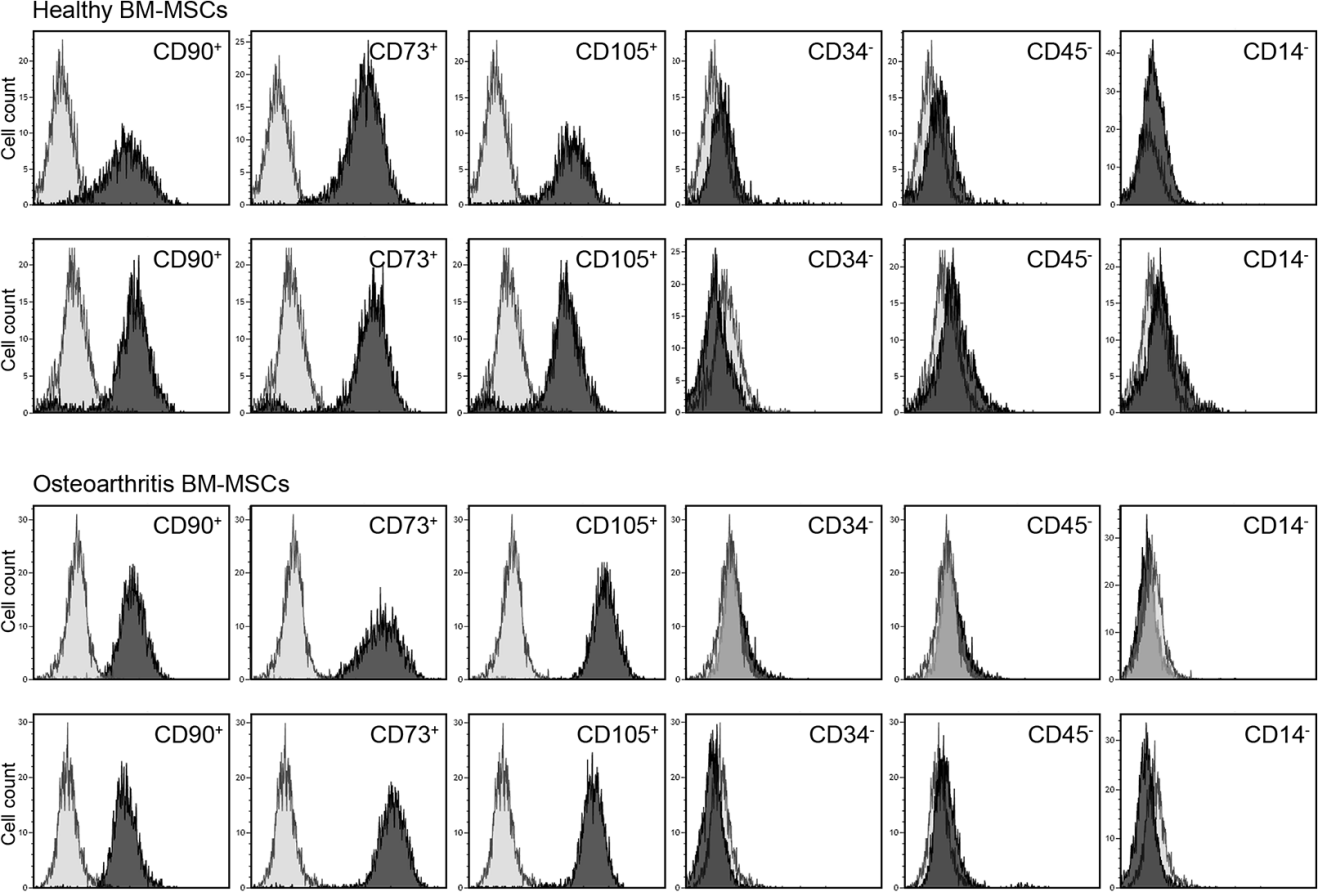
Characterization of MSCs by Flow Citometry. Representative flow Cytometry analysis of MSCs obtained from healthy subjects and OA Patients. MSCs at third passage were positive for MSC specific markers (CD90, CD73, CD105 and negative for CD34, CD45 and CD14). Figure represents an overlayed image of antibody isotype controls (light gray histogram) and specific marker antibodies (dark gray histogram). Healthy MSCs and OA-MSCs did not exhibit quantitative difference in terms of expression of these individual markers.

**Fig 2:**
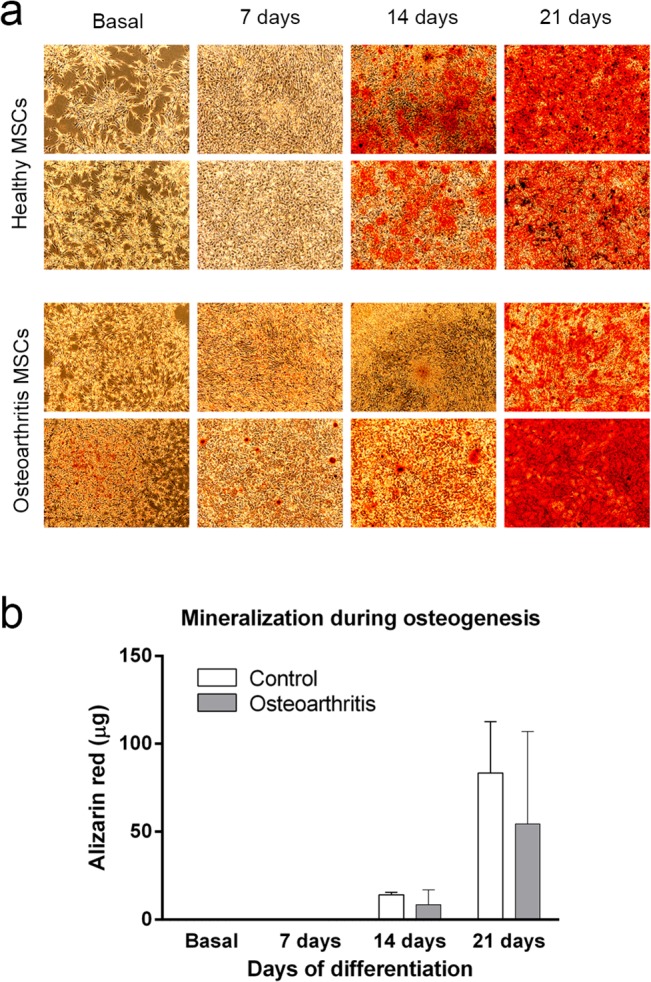
In vitro potential of differentiation of Healthy and OA-MSCs. a) *In vitro* differentiation and characterization of MSCs. Cultured MSCs from two healthy donors and two OA donors showing similar, plate adherence and fibroblast-like morphology at passage 3. Growth characteristics of MSCs from OA donors were similar as well as the degree of mineralization during osteogenic lineage commitment revealed by Alizarin red S staining (Panel A). (Magnification x 50). b) quantitative measurement of alizarin red staining of osteogenic cultures.

The phenotypic uniformity of MSCs used in the study were characterized according to, these criteria: the plastic-adherence in culture, the positive expression of CD90, CD73, CD105 absence of CD34, CD45 and CD14 expression and their osteogenic and chondrogenic lineage potential. Expression of positive and negative markers was similar for OA-MSCs and healthy-MSCs (Fig 1). Regardless of the group they belonged MSCs were able to differentiate to the osteogenic lineage (Fig 2). We did not find differences in growth rate and survival related to pathology.

### Gene Expression of Wnt Signaling Components during Osteogenic Differentiation

**Fig 3:**
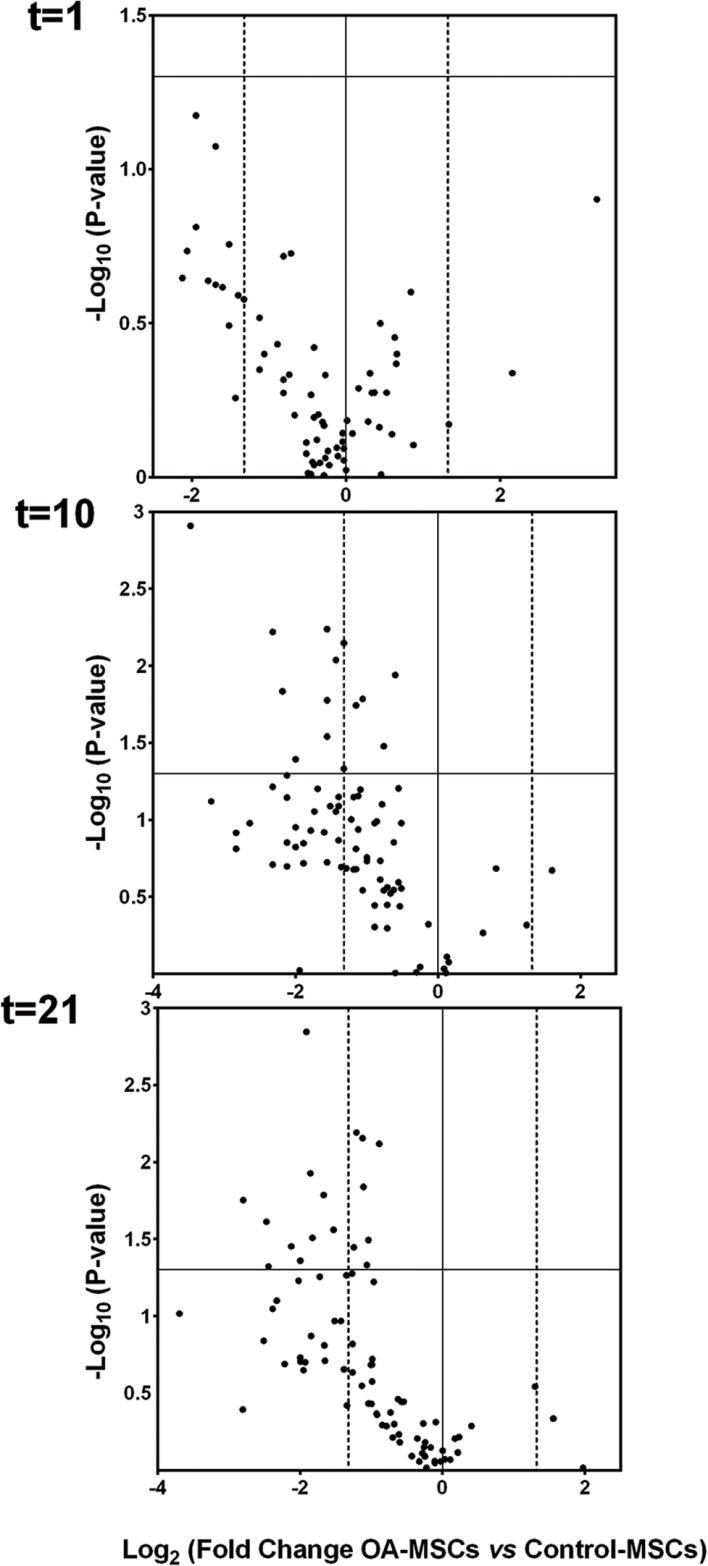
In vitro potential of differentiation of Healthy and OA-MSCs. Volcano plots representing the differential fold regulation in OA-MSCs compared to Healthy-MSCs at t = 1, 10 and 21 days of osteogenic differentiation. 84 WNT signaling pathway related genes analyzed in our study are represented. Vertical dashed lines indicate the 2.5-fold regulation cutoff. Significative genes with fold change <-2.5 and p-values <0.05 were seen only at days 10 and 21 and are indicated in S2 Table.

The expression of 84 genes involved in the Wnt pathway was analyzed during induced osteogenic differentiation of MSCs (S3 Table). In general and considering a 2.5 fold-change cutoff, differences between Healthy and OA-MSCs, a higher number of genes were downregulated in OA-MSCs throughout osteogenesis (Table 1, Fig 3 and S2 Table). A more detailed analysis at different time points revealed that no statistical differences were noticeable one day after osteogenic induction (t = 1), in particular with regard to the number of downregulated genes in OA-MSCs. However, this trend significantly changed during osteogenic differentiation, as revealed the dramatic increase in the number of differentially downregulated genes in OA-MSCs. Thus, at day 10 (t = 10) initially overexpressed *PITX2* reduced its overexpression and other additional 35 genes were clearly downregulated, 9 of which (*WNT5A*, *WNT5B*, *FZD4*, *LRP5*, *DVL1*, *PPP2CA*, *CSNK1G1*, *CSNK1A1* and *CSNK1D*) were significantly downregulated (S2 Table). Likewise, this trend was preserved until day 21 (t = 21) of differentiation, where a total of 30 genes were downregulated, ten of which significantly downregulated (*WNT2B*, *WNT5B*, *WNT9A*, *DKK1*, *FZD6*, *FZD3*, *DVL1*, *PPP2CA*, *CSNK1G1* and *PYGO1*) (Table 1 and S2 Table).These experiments showed a remarkably different gene expression pattern of Wnt related genes in OA-MSCs compared with Healthy-MSCs.

**Table 1:** Significant genes with fold change <±2.5 and p-values <0.05.

**Genes Over-Expressed in OA-MScs (t = 10)**			**Genes Under-Expressed in OA-MSCs (t = 10)**		
**Gene Symbol**	**Fold Regulation**	**p-value**	**Gene Symbol**	**Fold Regulation**	**p-value**
*PITX2*	*3*.*0272*	0.213282	***WNT5A***	**-10.6442**	**0.001233**
			***FZD4***	**-2.9066**	**0.005778**
			***WNT5B***	**-5.0118**	**0.006024**
	****		***PPP2CA***	**-2.6995**	**0.009167**
			***LRP5***	**-4.6161**	**0.014636**
			***CSNK1A1***	**-2.9759**	**0.016738**
			***DVL1***	**-2.9039**	**0.028821**
			***CSNK1G1***	**-4.0074**	**0.040399**
			***CSNK1D***	**-2.5198**	**0.046491**
**Genes Over-Expressed in OA-MScs (t = 21)**			**Genes Under-Expressed in OA-MSCs (t = 21)**		
**Gene Symbol**	**Fold Regulation**	**p-value**	**Gene Symbol**	**Fold Regulation**	**p-value**
*LEF1*	2.9404	0.462654	***WNT5B***	**-3.7616**	**0.001425**
*CSNK1A1*	3.9267	0.965739	***WNT9A***	**-3.6184**	**0.011803**
			***DVL1***	**-3.1807**	**0.016366**
			***PPP2CA***	**-6.9515**	**0.017637**
			***PYGO1***	**-5.5481**	**0.024419**
			***FZD6***	**-2.8865**	**0.027494**
			***FZD3***	**-3.5472**	**0.031007**
			***WNT2B***	**-4.355**	**0.035229**
			***CSNK1G1***	**-3.9852**	**0.043664**
			***DKK1***	**-5.4415**	**0.047558**

### Post-Translational Phosphorylation Occurring during Osteogenic Differentiation of MSCs

To establish the relationship between Wnt pathway activity and other molecular pathways, we investigated the status of several Receptor Tyrosine Kinases (RTKs) involved in downstream signaling cascades controlling basic cellular functions such as division, growth, metabolism, differentiation, migration and survival (S1Table).

**Fig 4:**
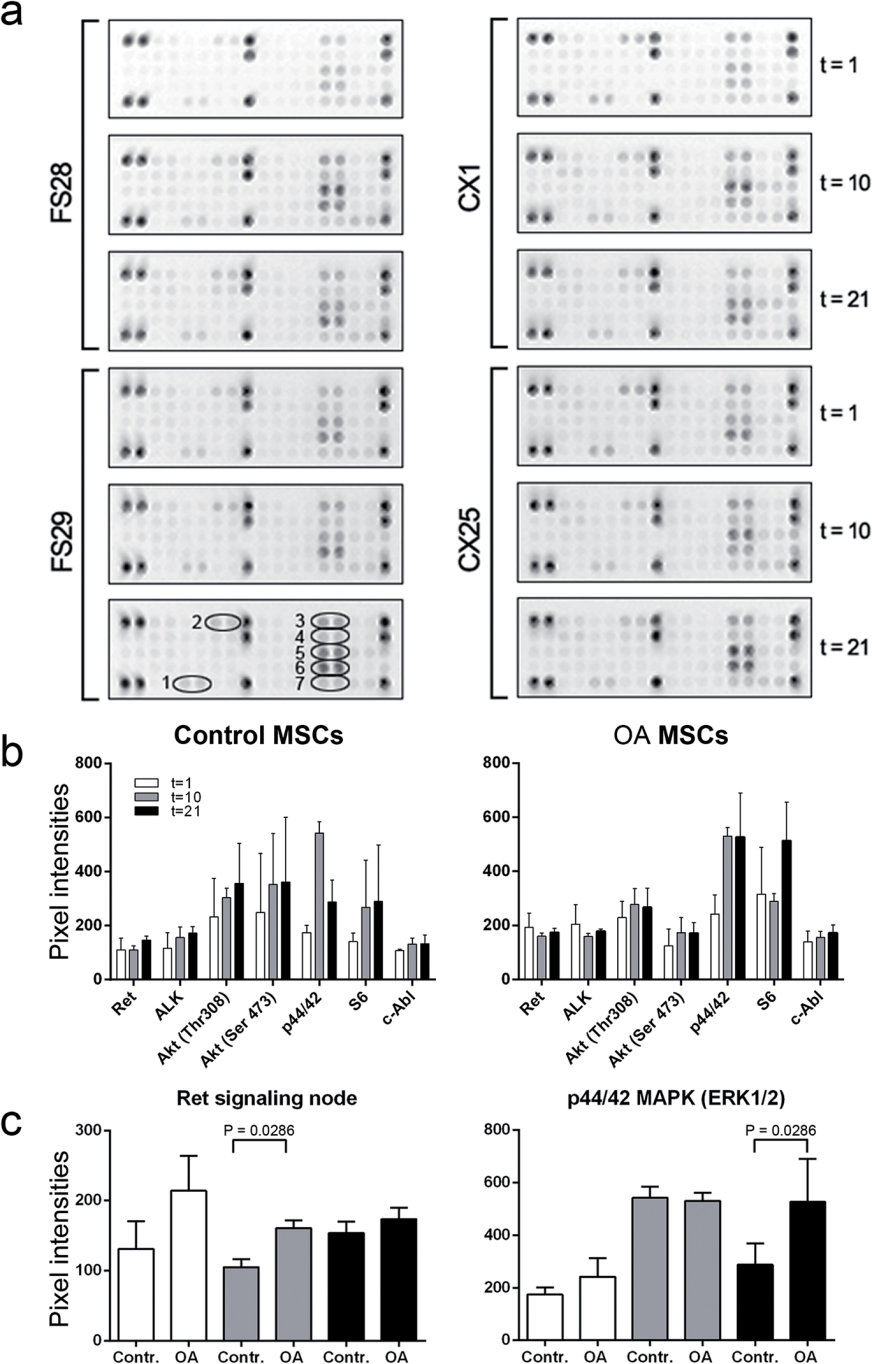
Phospho-protein array of Healthy-MSCs and OA-MSCs during osteogenesis. (a) Phospho-protein array of MSCs from two healthy (FS28 and FS29) and two OA individuals (CX1 and CX25) during osteogenic differentiation measured at days 1, 10 and 21. Image shows the phosphorylation status of 28 spotted duplicates for receptor tyrosine kinases (RTKs) and 11 signaling nodes where signal generated at each array spot is proportional to the amount of phospho-protein bound by each capture antibody. (b) Representation of the averaged pixel intensity values for the most prominent signals after background correction and further transformation as relative signal compared with the maximum signal of 10 positive controls (darker spots). 1: Ret Pan-Tyr. 2: Pan-Tyr ALK. 3: Akt/PKB/Rac at Thr308. 4: Akt/PKB/Rac at Ser473, 5. p44/42 MAPK (ERK1/2) at Thr202/Tyr204. 6: S6 Ribosomal Protein at Ser235/236 and 7: c-Abl (Pan-Tyr). (c) Significant differences in signal intensities between Healthy and OA MSCs were detected for phosphorylation signals of Ret Pan-Tyr at day 10 and p44/42 MAPK (ERK1/2) at day 21.

The phosphorylation status of various phosphorylated RTKs and signaling nodes was evaluated throughout osteogenesis. (Fig 4A) shows the existence of a similar phosphorylation pattern between healthy and OA-MSCs. Many RTKs did not show a significant signal over background, with the exception of Ret and ALK. Regarding the phosphorylation status of the signaling nodes, chemiluminescent signals were clearly positive for Akt/PKB/Rac at Thr308 and Ser473, for p44/42 MAPK (ERK1/2) S6 Ribosomal Protein at Ser235/236 and for c-Abl (Fig 4B). Significant differences in signal intensities between Healthy and OA MSCs were detected only at final stages of induced osteogenesis for Ret RTK and for p44/42 MAPK (ERK1/2) signaling node at final stages (Fig 4C).

**Fig 5:**
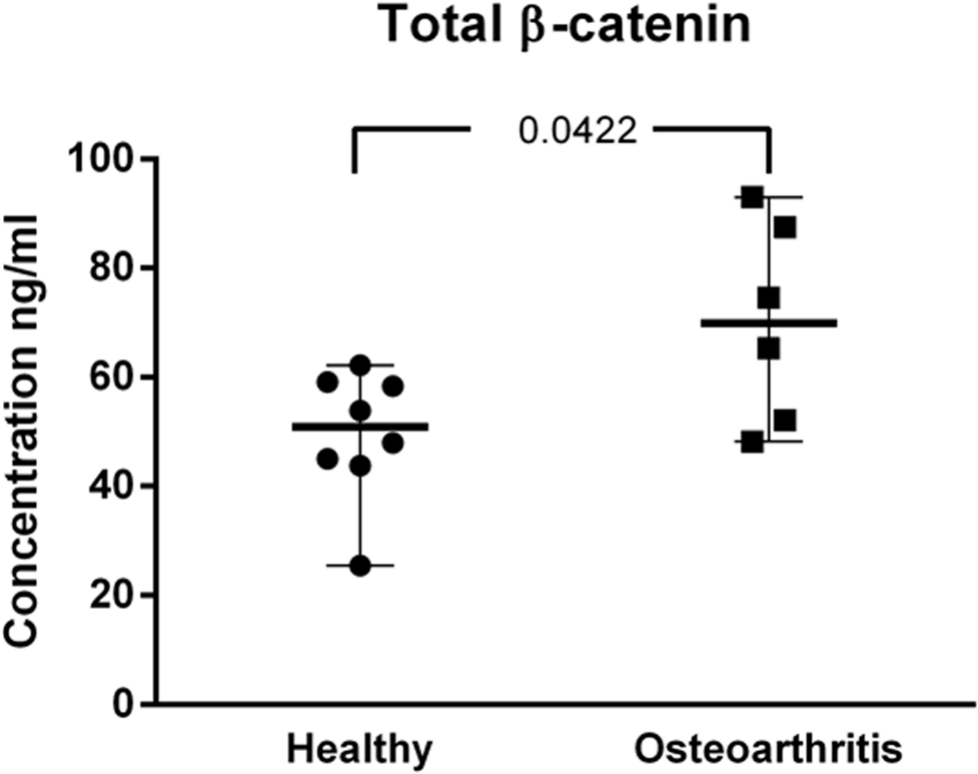
Total β-Catenin Enzyme-Linked ImmunoSorbent Assay (ELISA). Protein lysates of MSCs (10μg) were used for measurement of total β-Catenin content. Values represent the median wit range of triplicate determinations for eight Healthy donors and and six OA-MSCs.

Although the phosphorylation status of the serine/threonine kinase glycogen synthase kinase-3β (GSK-3β), that phosphorylates β-catenin targeting its degradation, was not included within these experiments, an ELISA was performed to determine the presence of total β-catenin. As shown in (Fig 5) levels of β-catenin were slightly increased in OA MSCs.

### Expression in MSCs of Osteogenesis Related Genes during Differentiation

During initiation and late mineralization of bone, a number of factors produced by osteogenic signaling pathways are recruited by RUNX2 to form a multicomponent transcriptional complex that interacts with the promoter regions of genes such as: alkaline phosphatase (ALP), osteopontin (OPN) or osteocalcin (BGLAP). RUNX2 thus, is a key transcription factor that determines the lineage commitment of MSCs to osteoblasts.

To determine the existence of differences in the expression of *RUNX2* and other genes regulated by RUNX2 during osteogenesis a quantitative PCR was performed to measure the relative expression levels of *RUNX2*, *ALP*, *OPN* and *BGLAP*.

**Fig 6:**
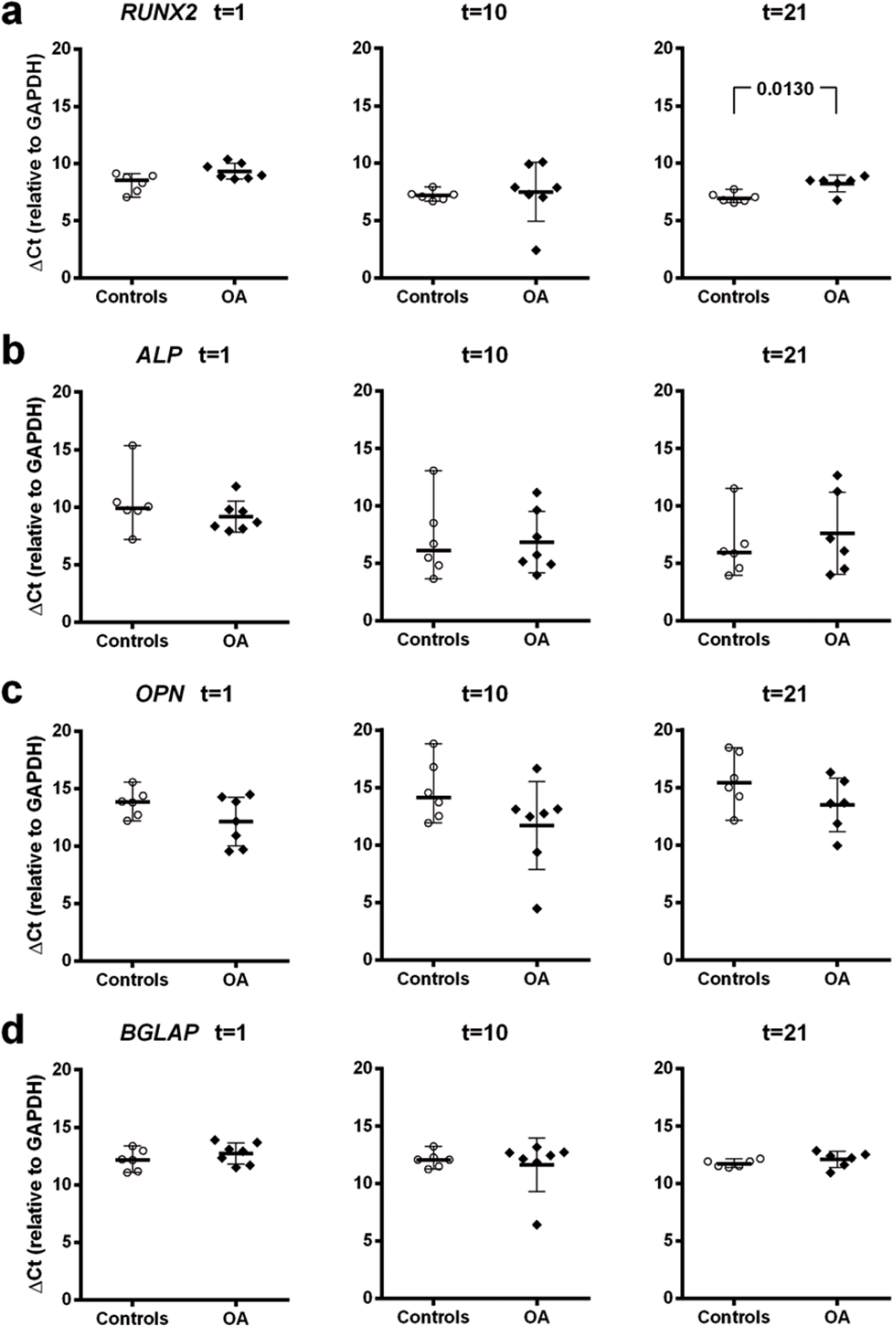
Expression of osteogenesis related genes during differentiation. Expression *in vitro* of *RUNX2*, *ALP* (Alkaline Phosphatase), *OPN* (Osteopontin) and *BGLAP* (Osteocalcin) in osteogenically induced cultures of Healthy-MSCs and OA-MSCs. Values represent de ΔCt values relative to expression of *GAPDH* refence gene.

Despite the existence of slight differences in expression between Healthy-MSCs and OA-MSCs relative to the expression of *RUNX2* at t = 21, expression of the other three genes analyzed were similar during induced osteogenesis (Fig 6).

## Discussion

Knowledge of the exact role of Wnt signaling and particularly its crosstalking with other pathways during MSCs differentiation, is still incomplete but essential to explain their involvement in OA initiation and/or progression, characterized not only by cartilage loss but also by changes in bone. Osteogenic differentiation of human MSCs from OA and healthy donors exhibited similar expression of membrane markers as well as osteogenic and chondrogenic lineage differentiation potentials under defined culture conditions. However, histochemical outcomes, based in qualitative differentiation assays, should be taken with caution as these assays might not display the status and role of particular pathway components. These drawbacks were partially addressed in this study performing a comparative and quantitative analysis of the Wnt signaling pathways during induced osteogenic differentiation. We reported the existence of clear differences between healthy and OA-MSCs, related to the expression of genes encoding components of the Wnt signaling pathway, such as secreted ligands, membrane receptors, co-receptors and intracellular and nuclear regulatory molecules. (S4 Table and S1 and S2 Figs)

Several members of the Wnt family of proteins signaling through the canonical and non-canonical pathways were significantly downregulated in OA-MSCs when differentiated to the osteogenic lineage. These include the genes *WNT2B*, *WNT9A*, *WNT5A* and *WNT5B*.

*WNT5A* and *WNT5B*, two classical non-canonical ligands described as activators or inhibitors of canonical Wnt signaling, depending on receptor context.[25] In the presence of the FZD4 transmembrane receptor, Wnt5A activates β-catenin signaling while in the presence of the ROR2 member of Ror-family of RTKs inhibits canonical Wnt signaling by promoting β-catenin degradation and down-regulation of β-catenin-induced gene expression through a GSK3-independent pathway, which involves down-regulation of β-catenin-induced reporter gene expression.[26] The importance of WNT5A crosstalking through the WNT5A/ROR2 axis has been described *in vitro* as crucial for enhancing bone resorption in skeletal homeostasis. [27, 28] However, *in vivo*, each Wnt can bind to many different Fz receptors and specificity may be restricted by temporal expression of both Wnt and Frizzled.[29] In our study OA-MSCs expression of frizzled receptors: *FZD3*, *FZD4*, *FZD6* and *LRP5* co-receptor were reduced. These results agree with *in vitro* studies demonstrating a reduced FZD6 expression in osteoblasts under mineralizing conditions.[30] Additionally, it has been described that, by unknown mechanisms, human FZD6 can inhibit Wnt/β-catenin signaling.[31] Bone mass regulation by LRPs was also demonstrated by loss-of-function and gain-of-function mutations in the LRP5 gene.[32, 33] Other functional studies also demonstrated that mutations in LRP5 result in a decreased binding of the Wnt signaling inhibitors sclerostin and dickkopf 1 (DKK1) with LRP5.[34–36] Collectively, although our findings suggest a lower activation of canonical Wnt signaling, specific ligand/receptor combinations in a particular cellular context can lead to different downstream events leading to an increased bone formation in OA. Here, we report the downregulation in OA-MSCs of *DVL1* and some casein kinases (CKs). The DVLs (DVL1, 2 and 3 in humans) are cytoplasmic proteins essential in both canonical and non-canonical Wnt pathways that participate by binding to the cytoplasmic C-terminus of frizzled family members and transduce the Wnt signal to down-stream effectors. Throughout this process PTMs, in particular phosphorylation mediated by CKs, are essential. It has been proposed that depending on Wnt stimulation and receptor context the sequential post-translational phosphorylation of DVLs by CKs, such as CK2 and PAR1 and Wnt inducible CK1δ/ε, can inhibit or activate the β-catenin destruction complex, controlling, in a negative feedback, the activation and the termination of the pathway.[37, 38] Similarly, CKs and *DVL1* downregulation seen in our study during OA-MSC osteogenesis, might be involved in a de-regulated control of the mechanisms mediated by DVL phosphorylation. Furthermore, DVLs are intermediary between Wnt-mediated activation of the Frizzled receptors and activation of CK2, the priming kinase of GSK-3β, which in turn modulates the function of β-catenin, c-myc, and c-Jun, targeting them for degradation or inactivation.[39] β-catenin priming at Ser45 by CK1 is also required for subsequent phosphorylation by GSK-3β.[40]

Given the redundant features of the genes analyzed, canonical and non-canonical pathways were differentially affected and thus likely the downstream developmental processes they regulate, in particular the cell fate, tissue polarity, cell growth, proliferation and migration (S4 Table). Post-translational phosphorylation and dephosphorylation are important mechanisms implicated in Wnt signaling modulation through crosstalking with other pathways. Our study showed the existence of a quite similar phosphorylation status between Control-MSCs and OA-MSCs of many phosphorylated proteins with the exception of Ret RTKs and the p44/42 MAPK (ERK1/2) signaling node. In addition to the moderate increase in *RUNX2* expression seen at early stages of osteoblast differentiation, this augmented post-translational phosphorylation agrees with their role during osteogenesis inducing the phosphorylation of RUNX2, and accumulation of other phosphorylated proteins.[41] These findings are also consistent with the *in vivo* description of an altered bidirectional signaling between subchondral bone osteoblasts and articular cartilage chondrocytes mediated by the phosphorylation of MAPK (ERK1/2) signaling pathway.[42] In addition to their well know function as mediators of inflammation, MAPKs are also signal transducers that regulate bone mass, controlling the expression of osteogenic genes and the proliferative activity and differentiation of osteoblasts, [43] this function in humans is mainly initiated by an increased activity after RUNX2 phosphorylation more than by a significant increase in protein or mRNA levels.[44]

The differences found, might function as modulators of Wnt signal intensity and vice versa. For example, downregulation of *PP2CA* could be involved in a lower dephosphorylation of signaling nodes, and consequently activating key signaling pathways.

Overinterpretation of gene expression data and the use of different MSCs for some experiments could be a major methodological limitation of the current study. Besides, the *in vitro* culture system and time windows used for assessment of osteogenesis could override subtle changes occurring during bone differentiation *in vivo*. However, we clearly reported a downregulation of genes within the Wnt pathway in OA-MSC but without a significant difference in their *in vitro* osteogenic potential. Moreover increased levels of β-catenin found in OA-MSCs does not correlate with an increased osteogenic potential of these cells likely suggesting the existence of compensatory mechanisms in OA-MSCs modulating their osteogenic transcriptional regulation. For example, translation and transcription could be regulated by divergent mechanisms involving proteins such as transcription factors and/or translational repression by small non-coding miRNAs. These, can alter the protein level translated from a given target mRNA without altering the amount of RNA. In this regard, overexpression of miR-335-5p has been described to enhance the osteogenic differentiation by specific downregulation of DKK1.[45]

As bone homeostasis is regulated by a large number of factors, including inflammatory factors present in OA that can induce different effects. A complete understanding of the MSC differentiation will require further *in vivo* studies. For example, in rheumatoid arthritis, inhibition of Wnt signaling has been associated with lack of repair whereas Wnts have been associated with the ankylosing phenotype in spondyloarthritis.[46, 47] Moreover, rare human mutations affecting bone negatively (low-bone mass phenotype, osteoporosis-pseudoglioma syndrome) or positively (sclerosteosis and Van Buchem disease) have been identified in components of the canonical Wnt signaling.[48, 49]

Collectively, these observations provide an approach to the intricate crosstalk between Wnt and other signaling pathways in osteoarthritis and recognize the Wnt pathway as an attractive target for future therapeutic intervention and manipulation of these signaling pathways to treat disorders with presence of a dysregulated osteogenesis, such as OA and other musculoskeletal diseases.

## Supporting Information

S1 Fig Color diagram of the human Wnt pathway. (PDF)

S2 Fig Known and predicted interactions between proteins coded by the genes downregulated in OA-MSCs. (PDF)

S1 Table Receptor Tyrosine Kinases (RTKs). (PDF)

S2 Table Fold change values of all genes analyzed. (PDF)

S3 Table List of the 84 genes analyzed. (PDF)

S4 Table WNT Pathway related genes and functions. (PDF)
