# Supplementary material for: Altered Expression of Wnt Signaling Pathway Components in Osteogenesis of Mesenchymal Stem Cells in Osteoarthritis Patients
Source: PLoS One. 2015 Sep 9;10(9):e0137170. doi: 10.1371/journal.pone.0137170 (PMC4564164; doi:10.1371/journal.pone.0137170)

Supporting information Image S5.
Diagram of the human Wnt pathway

Diagram based on the human Wnt pathway modified from the KEGG (Kyoto enciclopedia of Genes and Genomes) map 04310. Genes analyzed and present in our array are indicated inside green text boxes.

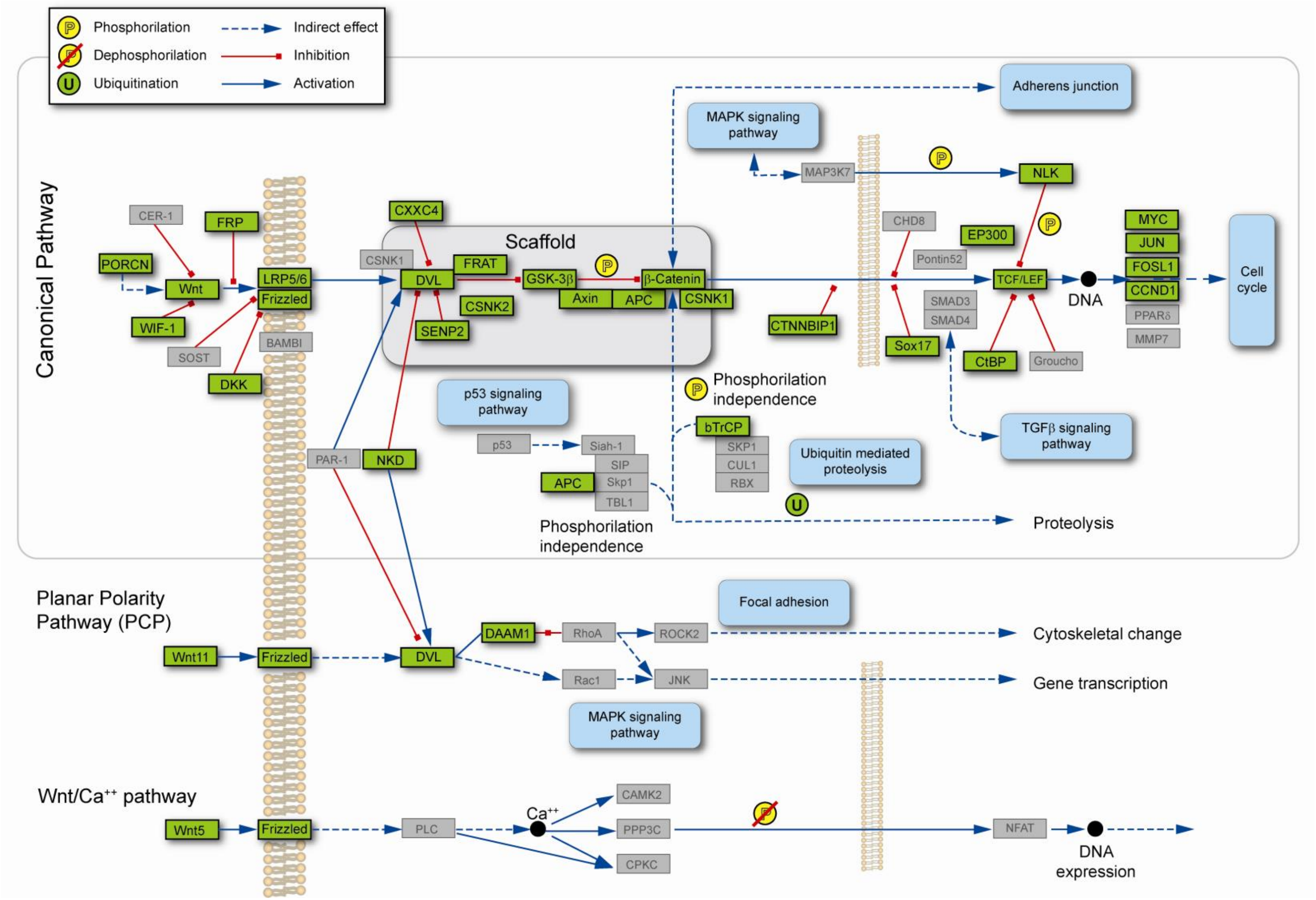

Supplement: S1 Fig — (PDF) [file pone.0137170.s001.pdf]
