# Supplementary material for: Altered Expression of Wnt Signaling Pathway Components in Osteogenesis of Mesenchymal Stem Cells in Osteoarthritis Patients
Source: PLoS One. 2015 Sep 9;10(9):e0137170. doi: 10.1371/journal.pone.0137170 (PMC4564164; doi:10.1371/journal.pone.0137170)

Supporting information Image S6.

Known and predicted interactions between proteins coded by the genes downregulated in OA-MSCs

Network Display of predicted functional links using the STRING 9.05 database of known and predicted protein interactions (http://string-db.org/). The image represents the confidence view interactions where the stronger associations are represented by thicker lines.

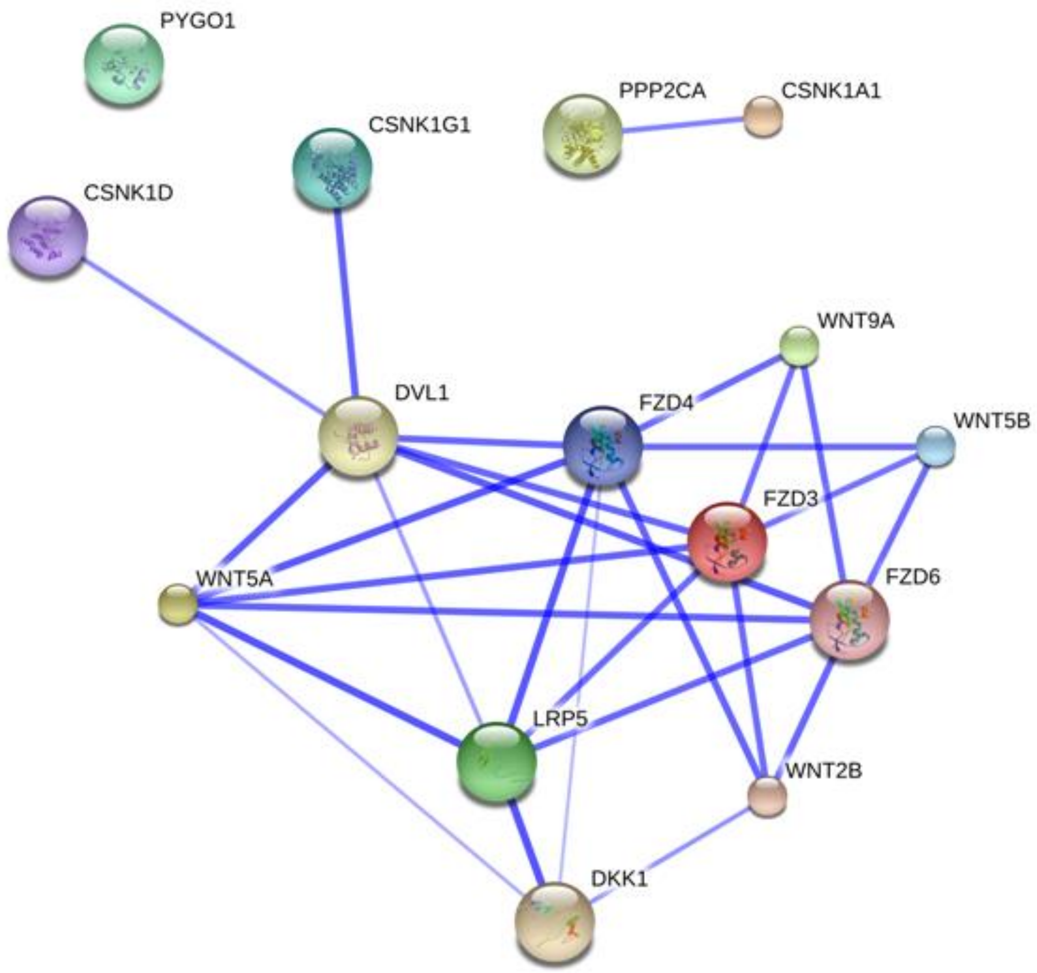

Supplement: S2 Fig — (PDF) [file pone.0137170.s002.pdf]
