# Supplementary material for: Altered Expression of Wnt Signaling Pathway Components in Osteogenesis of Mesenchymal Stem Cells in Osteoarthritis Patients
Source: PLoS One. 2015 Sep 9;10(9):e0137170. doi: 10.1371/journal.pone.0137170 (PMC4564164; doi:10.1371/journal.pone.0137170)

Supporting information Table S1.
Receptor Tyrosine Kinases (RTKs)

Target	Phosphorylation Site	Family of receptors
**1:** EGFR/ErbB1<br>**2:** HER2/ErbB2<br>**3:** HER3/ErbB3	pan-Tyr	Epidermal Growth Factor Receptor (EGFR)
**4:** FGFR1<br>**5:** FGFR3<br>**6:** FGFR4	pan-Tyr	Fibroblast Growth Factor Receptor (FGFR)
**7:** InsR<br>**8:** IGF-IR	pan-Tyr	Insulin Receptor
**9:** TrkA/NTRK1<br>**10:** TrkB/NTRK2	pan-Tyr	Nerve growth factor receptor (NGFR)
**11:** Met/HGFR<br>**12:** Ron/MST1R	pan-Tyr	Hepatocyte Growth Factor Receptor (HGFR)
**13:** Ret	pan-Tyr	Ret (RET receptor family)
**14:** ALK	pan-Tyr	Lymphocyte-specific Tyrosine Kinase (LTK)
**15:** PDGFR<br>**16:** c-Kit/SCFR<br>**17:** FLT3/Flk2<br>**18:** M-CSFR/CSF-1R	pan-Tyr	Platelet-Derived Growth Factor Receptor (PDGFR)
**19:** EphA1<br>**20:** EphA2<br>**21:** EphA3<br>**22:** EphB1<br>**23:** EphB3<br>**24:** EphB4	pan-Tyr	Ephrins related Receptors (EphR)
**25:** Tyro3/Dtk<br>**26:** Axl	pan-Tyr	Axl
**27:** Tie2/TEK	pan-Tyr	Tie
**28:** VEGFR2/KDR	pan-Tyr	VEGFR

Signaling Nodes

Target	Phosphorylation Site	Family
**29:** Akt/PKB/Rac<br>**30:** Akt/PKB/Rac	Thr308<br>Ser473	Akt
**31:** p44/42 MAPK (ERK1/2)	Thr202/Tyr204	MAPK
**32:** S6 Ribosomal Protein	Ser235/236	RSK
**33:** c-Abl	pan-Tyr	Abl
**34:** IRS-1	pan-Tyr	IRS
**35:** Zap-70	pan-Tyr	Zap-70
**36:** Src<br>**37:** Lck	pan-Tyr<br>pan-Tyr	Src
**38:** Stat1<br>**39:** Stat3	Tyr701<br>Tyr705	Stat

Target map of the Array Kit

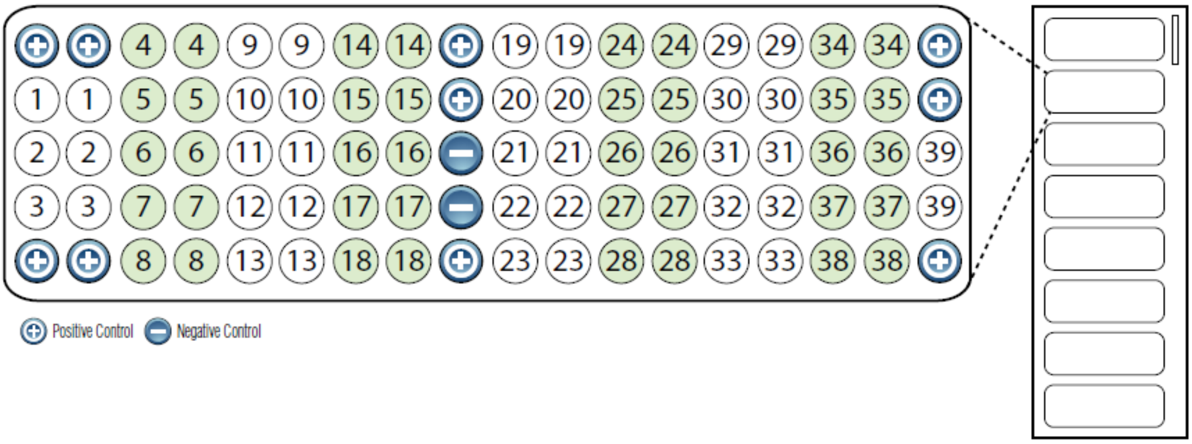

Supplement: S1 Table — (PDF) [file pone.0137170.s003.pdf]
