# Supplementary material for: Altered Expression of Wnt Signaling Pathway Components in Osteogenesis of Mesenchymal Stem Cells in Osteoarthritis Patients
Source: PLoS One. 2015 Sep 9;10(9):e0137170. doi: 10.1371/journal.pone.0137170 (PMC4564164; doi:10.1371/journal.pone.0137170)
Supplement: S2 Table — (PDF) [file pone.0137170.s004.pdf]

Supporting information Table S2.

Significant genes with fold change <±2.5 and p-values <0.05 during osteogenesis are indicated in bold.

Genes Over-Expressed in OA-MScs (t=1)

Gene Symbol	Fold Regulation	p-value
PITX2	9.5137	0.125297
FZD4	4.4527	0.458919
CTNNBIP1	2.5233	0.673767

Genes Under-Expressed in OA-MSCs (t=1)

Gene Symbol	Fold Regulation	p-value
TLE2	-3.8229	0.066967
WNT2B	-3.255	0.084368
WNT11	-3.8852	0.154081
CXXC4	-2.8639	0.175365
WNT9A	-4.122	0.184401
FSHB	-4.4158	0.225592
WNT7A	-3.4806	0.230234
WNT10A	-3.2043	0.236967
WNT8A	-3.0035	0.241633
WNT7B	-2.6268	0.256884
WNT1	-2.514	0.264391
CCND2	-2.8959	0.321531
WNT2	-2.7346	0.553172

Genes Over-Expressed in OA-MScs (t=10)

Gene Symbol	Fold Regulation	p-value
PITX2	*3.0272*	0.213282

Genes Under-Expressed in OA-MSCs (t=10)

Gene Symbol	Fold Regulation	p-value
WNT5A	**-10.6442**	**0.001233**
FZD4	**-2.9066**	**0.005778**
WNT5B	**-5.0118**	**0.006024**
PPP2CA	**-2.6995**	**0.009167**
LRP5	**-4.6161**	**0.014636**
CSNK1A1	**-2.9759**	**0.016738**
DVL1	**-2.9039**	**0.028821**
CSNK1G1	**-4.0074**	**0.040399**
CSNK1D	**-2.5198**	**0.046491**
DKK1	-4.3489	0.051449
WNT7A	-5.0583	0.061087
FGF4	-3.252	0.062916
T	-2.6123	0.070974
WNT2B	-4.2693	0.071714
FZD3	-9.0005	0.075898
FZD6	-2.8232	0.081395
CTNNB1	-2.6184	0.081472
TLE1	-2.6945	0.088335
NKD1	-3.2807	0.088425
TLE2	-6.196	0.105323
WNT11	-3.9632	0.111832
FZD5	-4.4239	0.140147
WIF1	-3.471	0.117381
WNT1	-3.0752	0.120467
FRZB	-7.3242	0.121359
DVL2	-2.6293	0.135906
FSHB	-3.7546	0.141999
WNT10A	-3.9322	0.150031
WNT8A	-7.1536	0.154164

SLC9A3R1	-2.9649	0.188583
SOX17	-3.7633	0.191632
SFRP4	-4.9611	0.195212
WNT3A	-4.3229	0.200618
WNT2	-2.5444	0.20276
FOXN1	-3.8655	0.951802

Genes Over-Expressed in OA-MScs (t=21)

Gene Symbol	Fold Regulation	p-value
LEF1	2.9404	0.462654
CSNK1A1	3.9267	0.965739

Genes Under-Expressed in OA-MSCs (t=21)

Gene Symbol	Fold Regulation	p-value
WNT5B	**-3.7616**	**0.001425**
WNT9A	**-3.6184**	**0.011803**
DVL1	**-3.1807**	**0.016366**
PPP2CA	**-6.9515**	**0.017637**
PYGO1	**-5.5481**	**0.024419**
FZD6	**-2.8865**	**0.027494**
FZD3	**-3.5472**	**0.031007**
WNT2B	**-4.355**	**0.035229**
CSNK1G1	**-3.9852**	**0.043664**
DKK1	**-5.4415**	**0.047558**
TLE1	-2.5456	0.054286
DVL2	-3.305	0.055439
FZD5	-4.0577	0.058843
WNT5A	-5.0164	0.079365
FOXN1	-5.2222	0.089814
FRZB	-12.9361	0.096201
FBXW4	-2.6858	0.107529
NKD1	-2.8573	0.107566
LRP5	-3.5917	0.134602
SFRP4	-5.6909	0.144795
WNT10A	-3.1587	0.155064
APC	-3.9871	0.186039
SOX17	-3.1398	0.194934
WNT11	-3.9816	0.197681
WNT16	-3.8018	0.199757
WNT3A	-4.6439	0.204744
SLC9A3R1	-2.6075	0.221278
TLE2	-3.8673	0.224486
CXXC4	-2.5315	0.380725
PPP2R1A	-6.9773	0.404535
