# Supplementary material for: Altered Expression of Wnt Signaling Pathway Components in Osteogenesis of Mesenchymal Stem Cells in Osteoarthritis Patients
Source: PLoS One. 2015 Sep 9;10(9):e0137170. doi: 10.1371/journal.pone.0137170 (PMC4564164; doi:10.1371/journal.pone.0137170)
Supplement: S3 Table — (PDF) [file pone.0137170.s005.pdf]

Supporting information Table S3. List of the 84 genes analyzed.

Symbol	Unigene	Refseq	Description
AES	Hs.515053	NM_001130	Amino-terminal enhancer of split
APC	Hs.158932	NM_000038	Adenomatous polyposis coli
AXIN1	Hs.592082	NM_003502	Axin 1
BCL9	Hs.415209	NM_004326	B-cell CLL/lymphoma 9
BTRC	Hs.643802	NM_033637	Beta-transducin repeat containing
FZD5	Hs.17631	NM_003468	Frizzled family receptor 5
CCND1	Hs.523852	NM_053056	Cyclin D1
CCND2	Hs.376071	NM_001759	Cyclin D2
CCND3	Hs.534307	NM_001760	Cyclin D3
CSNK1A1	Hs.529862	NM_001892	Casein kinase 1, alpha 1
CSNK1D	Hs.631725	NM_001893	Casein kinase 1, delta
CSNK1G1	Hs.646508	NM_022048	Casein kinase 1, gamma 1
CSNK2A1	Hs.644056	NM_001895	Casein kinase 2, alpha 1 polypeptide
CTBP1	Hs.208597	NM_001328	C-terminal binding protein 1
CTBP2	Hs.501345	NM_022802	C-terminal binding protein 2
CTNNB1	Hs.476018	NM_001904	Catenin (cadherin-associated protein), beta 1, 88kDa
CTNNBIP1	Hs.463759	NM_020248	Catenin, beta interacting protein 1
CXXC4	Hs.12248	NM_025212	CXXC finger protein 4
DAAM1	Hs.654934	NM_014992	Dishevelled associated activator of morphogenesis 1
DIXDC1	Hs.655626	NM_033425	DIX domain containing 1
DKK1	Hs.40499	NM_012242	Dickkopf homolog 1 (Xenopus laevis)
DVL1	Hs.74375	NM_004421	Dishevelled, dsh homolog 1 (Drosophila)
DVL2	Hs.118640	NM_004422	Dishevelled, dsh homolog 2 (Drosophila)
EP300	Hs.517517	NM_001429	E1A binding protein p300
FBXW11	Hs.484138	NM_012300	F-box and WD repeat domain containing 11
FBXW2	Hs.494985	NM_012164	F-box and WD repeat domain containing 2
FGF4	Hs.1755	NM_002007	Fibroblast growth factor 4
FOSL1	Hs.283565	NM_005438	FOS-like antigen 1
FOXN1	Hs.663679	NM_003593	Forkhead box N1
FRAT1	Hs.126057	NM_005479	Frequently rearranged in advanced T-cell lymphomas
FRZB	Hs.128453	NM_001463	Frizzled-related protein
FSHB	Hs.36975	NM_000510	Follicle stimulating hormone, beta polypeptide
FZD1	Hs.94234	NM_003505	Frizzled family receptor 1
FZD2	Hs.142912	NM_001466	Frizzled family receptor 2
FZD3	Hs.40735	NM_017412	Frizzled family receptor 3
FZD4	Hs.19545	NM_012193	Frizzled family receptor 4
FZD6	Hs.591863	NM_003506	Frizzled family receptor 6
FZD7	Hs.173859	NM_003507	Frizzled family receptor 7
FZD8	Hs.302634	NM_031866	Frizzled family receptor 8
GSK3A	Hs.466828	NM_019884	Glycogen synthase kinase 3 alpha
GSK3B	Hs.445733	NM_002093	Glycogen synthase kinase 3 beta

Symbol	Unigene	Refseq	Description
JUN	Hs.714791	NM_002228	Jun proto-oncogene
KREMEN1	Hs.229335	NM_001039570	Kringle containing transmembrane protein 1
LEF1	Hs.555947	NM_016269	Lymphoid enhancer-binding factor 1
LRP5	Hs.6347	NM_002335	Low density lipoprotein receptor-related protein 5
LRP6	Hs.584775	NM_002336	Low density lipoprotein receptor-related protein 6
MYC	Hs.202453	NM_002467	V-myc myelocytomatosis viral oncogene homolog (avian)
NKD1	Hs.592059	NM_033119	Naked cuticle homolog 1 (Drosophila)
NLK	Hs.208759	NM_016231	Nemo-like kinase
PITX2	Hs.643588	NM_000325	Paired-like homeodomain 2
PORCN	Hs.386453	NM_022825	Porcupine homolog (Drosophila)
PPP2CA	Hs.483408	NM_002715	Protein phosphatase 2, catalytic subunit, alpha isozyme
PPP2R1A	Hs.467192	NM_014225	Protein phosphatase 2, regulatory subunit A, alpha
PYGO1	Hs.256587	NM_015617	Pygopus homolog 1 (Drosophila)
RHOU	Hs.647774	NM_021205	Ras homolog gene family, member U
SENP2	Hs.401388	NM_021627	SUMO1/sentrin/SMT3 specific peptidase 2
SFRP1	Hs.713546	NM_003012	Secreted frizzled-related protein 1
SFRP4	Hs.658169	NM_003014	Secreted frizzled-related protein 4
FBXW4	Hs.500822	NM_022039	F-box and WD repeat domain containing 4
SLC9A3R1	Hs.728760	NM_004252	Solute carrier family 9 (sodium/hydrogen exchanger)
SOX17	Hs.98367	NM_022454	SRY (sex determining region Y)-box 17
T	Hs.389457	NM_003181	T, brachyury homolog (mouse)
TCF7	Hs.573153	NM_003202	Transcription factor 7 (T-cell specific, HMG-box)
TCF7L1	Hs.516297	NM_031283	Transcription factor 7-like 1 (T-cell specific, HMG-box)
TLE1	Hs.197320	NM_005077	Transducin-like enhancer of split 1
TLE2	Hs.332173	NM_003260	Transducin-like enhancer of split 2
WIF1	Hs.284122	NM_007191	WNT inhibitory factor 1
WISP1	Hs.492974	NM_003882	WNT1 inducible signaling pathway protein 1
WNT1	Hs.248164	NM_005430	Wingless-type MMTV integration site family, member 1
WNT10A	Hs.121540	NM_025216	Wingless-type MMTV integration site family, member 10A
WNT11	Hs.108219	NM_004626	Wingless-type MMTV integration site family, member 11
WNT16	Hs.272375	NM_057168	Wingless-type MMTV integration site family, member 16
WNT2	Hs.567356	NM_003391	Wingless-type MMTV integration site family member 2
WNT2B	Hs.258575	NM_004185	Wingless-type MMTV integration site family, member 2B
WNT3	Hs.445884	NM_030753	Wingless-type MMTV integration site family, member 3
WNT3A	Hs.336930	NM_033131	Wingless-type MMTV integration site family, member 3A
WNT4	Hs.25766	NM_030761	Wingless-type MMTV integration site family, member 4
WNT5A	Hs.696364	NM_003392	Wingless-type MMTV integration site family, member 5A
WNT5B	Hs.306051	NM_032642	Wingless-type MMTV integration site family, member 5B
WNT6	Hs.29764	NM_006522	Wingless-type MMTV integration site family, member 6
WNT7A	Hs.72290	NM_004625	Wingless-type MMTV integration site family, member 7A
WNT7B	Hs.512714	NM_058238	Wingless-type MMTV integration site family, member 7B
WNT8A	Hs.591274	NM_058244	Wingless-type MMTV integration site family, member 8A
WNT9A	Hs.149504	NM_003395	Wingless-type MMTV integration site family, member 9A
RPL13A	**Hs.728776**	**NM_012423**	**Ribosomal protein L13a (Housekeeping gene)**
