# Supplementary material for: Altered Expression of Wnt Signaling Pathway Components in Osteogenesis of Mesenchymal Stem Cells in Osteoarthritis Patients
Source: PLoS One. 2015 Sep 9;10(9):e0137170. doi: 10.1371/journal.pone.0137170 (PMC4564164; doi:10.1371/journal.pone.0137170)
Supplement: S4 Table — (PDF) [file pone.0137170.s006.pdf]

Supporting information Table S4.

WNT Pathway related genes and functions

Canonical Wnt

AES (TLE/Groucho), APC, AXIN1,, BCL9, CSNK1A1, CSNK1D, CSNK1G1, CSNK2A1, CTBP1, CTBP2, CTNNB1, CTNNBIP1 (ICAT), CXXC4, DIXDC1, DKK1, DVL1, DVL2, EP300, FRAT1, FZD1, FZD2, FZD3, FZD4, FZD5, FZD6, FZD7, FZD8, GSK3A, GSK3B, LEF1, LRP5, LRP6, NKD1, PORCN, PPP2CA, PPP2R1A, PYGO1, SENP2, SFRP1, SFRP4, SOX17, TCF7, TCF7L1, WIF1, WNT1, WNT10A, WNT16, WNT2, WNT2B, WNT3, WNT3A, WNT4, WNT6, WNT7A, WNT7B, WNT8A.

Planar Cell Polarity (PCP)

DAAM1, DVL1, DVL2, NKD1, RHOU, WNT9A.

WNT/Ca++

FZD2, WNT1, WNT10A, WNT11, WNT16 WNT2, WNT2B, WNT3, WNT3A, WNT4, WNT5A, WNT5B WNT6, WNT7A, WNT7B, WNT8A WNT9A.

WNT Signaling Negative Regulation

APC, AXIN1, BTRC (bTrCP), CCND1, CTBP1, CTBP2, CTNNBIP1 (ICAT), CXXC4, DKK1, FBXW11, FBXW2, FBXW4, FRZB (FRP-3), KREMEN1, LRP6, NLK, NKD1, SENP2, SFRP1, SFRP4, SOX17, TLE1, TLE2, WIF1.

Signaling Target Genes

BTRC (bTrCP), CCND1, CCND2, CCND3, FOSL1 (FRA-1), JUN, MYC, PITX2, T (Brachyury)

Cell Fate

CTNNB1, DKK1, WNT1L,WNT3 WNT3A

Tissue Polarity

FZD2, FZD3, FZD5, FZD6

Cell Growth & Profileration

APC, CCND1, CCND2, CCND3, CTBP1, CTBP2, CTNNB1, CTNNBIP1(ICAT), EP300, FGF4, FOSL1, FOXN1, FSHB, FZD3, JUN, LRP5, MYC, PPP2CA, PPP2R1A, T (Brachyury), WISP1, WNT3A.

Cell Migration

APC, DKK1, LRP5, LRP6, WNT1

Cell Cycle

APC, BTRC(bTrCP), CCND1, CCND2, CCND3, CTNNB1, EP300, FOSL1, JUN, MYC, RHOU, TCF7L1

Cellular Homeostasis
APC, FZD2, JUN, MYC, SLC9A3R1
